# Telomere length as a predictor of response to Pioglitazone in patients with unremitted depression: a preliminary study

**DOI:** 10.1038/tp.2015.187

**Published:** 2016-01-05

**Authors:** N Rasgon, K W Lin, J Lin, E Epel, E Blackburn

**Affiliations:** 1 Department of Psychiatry and Behavioral Sciences, Stanford University School of Medicine, Stanford, CA, USA; 2 Department of Biochemistry and Biophysics, University of California, San Francisco, San Francisco, CA, USA; 3 Department of Psychiatry, University of California, San Francisco, San Francisco, CA, USA

## Abstract

We studied peripheral leukocyte telomere length (LTL) as a predictor of antidepressant response to PPAR-γ agonist in patients with unremitted depression. In addition we examined correlation between LTL and the insulin resistance (IR) status in these subjects. Forty-two medically stable men and women ages 23–71 with non-remitted depression participated in double-blind placebo-controlled add-on of Pioglitazone to treatment-as-usual. Oral glucose tolerance tests were administered at baseline and at 12 weeks. Diagnostic evaluation of psychiatric disorders was performed at baseline and mood severity was followed weekly throughout the duration of the trial. At baseline, no differences in LTL were detected by depression severity, duration or chronicity. LTL was also not significantly different between insulin-resistant and insulin-sensitive subjects at baseline. Subjects with longer telomeres exhibited greater declines in depression severity in the active arm, but not in a placebo arm, *P*=0.005, *r*=−0.63, 95% confidence interval (95% CI)=(−0.84,−0.21). In addition, LTL predicted improvement in insulin sensitivity in the group overall and did not differ between intervention arms, *P*=0.036, *r*=−0.44, 95% CI=(−0.74,0.02) for the active arm, and *P*=0.026, *r*=−0.50, 95% CI=(−0.78,−0.03) for the placebo arm. LTL may emerge as a viable predictor of antidepressant response. An association between insulin sensitization and LTL regardless of the baseline IR status points to potential role of LTL as a non-specific moderator of metabolic improvement in these patients.

## Introduction

Leukocyte telomere length (LTL) provides an index of cellular aging that can predict incidence of age-related diseases, such as cardiovascular disease (CVD) and type 2 diabetes.^1, 2, 3, 4, 5, 6^ Insulin resistance (IR), a highly prevalent metabolic abnormality, significantly increases risk for CVD and type 2 diabetes.^7, 8^ Previous studies have described associations between shortened telomeres and depression,^9, 10^ as well as chronic mood and anxiety disorders^11^ although not all studies support the possibility that LTL is a predictor of depression.

Depressive disorders are among most prevalent psychiatric illnesses and are highly co-morbid with CVDs, diabetes among others.^12,13,14^ While individual prevalence for both IR and depression are rapidly increasing,^12,13^ the non-random co-occurrence of IR and depression is striking.^14^ Yet the understanding of this relationship is limited. Following our early postulate that IR is a part of—and may contribute to—the pathophysiology of depression,^15, 16^ we reported an antidepressant response to an insulin-sensitizing agent, metformin,^17^ and to the PPAR-γ agonists rosiglitazone^18^ and Pioglitazone^19^ in patients with unremitted depression.

In addition to the metabolic dysfunction of IR, when compared with non-depressed individuals, patients with major depression have been found to exhibit all of the cardinal features of inflammation.^20, 21^ Because increased inflammatory markers at baseline predict an antidepressant response,^22, 23, 24^ reducing inflammation may also augment response to psychotropic medications. Few studies to date have investigated an association between LTL and therapeutic response in depression, reporting both presence^25^ and absence^26^ of such an association. No studies, however, assessed LTL and response to treatment with PPAR-γ agonists. This report is a part of parent study (under review) of an antidepressant response to an adjuvant PPAR-γ agonist, Pioglitazone, in patients with unremitted depression.^19^ Here we aimed to assess LTL as a predictor of antidepressant response to Pioglitazone in groups of IR and insulin-sensitive (IS) subjects using surrogate markers of IR.

## Materials and methods

The Stanford University Institutional Review Board approved the current study in its entirety. All participants provided informed consent prior to study enrollment after they received detailed information regarding all study procedures, potential side effects of study medication, risks and benefits of participation, and contact personnel in case of questions or concerns. Study participants were recruited through collaboration with established investigators in the area of adult depression within the Department of Psychiatry & Behavioral Sciences at Stanford University, as well as community health providers. In addition, participants were recruited through advertisements in local newspapers and clinical trial recruitment websites.

Inclusion criteria included being between 21 and 75-years-old, having a body mass index of 18.5 to 40 kg/m^2^, having at least 12 years of education, having a history of depression with at least 8 weeks of stable treatment-as-usual for depression. We did not exclude subjects on a basis of a type of medication(s) used in treatment of depression, as our primary goal was to evaluate antidepressant qualities of adjunctive Pioglitazone.

Exclusion criteria included a history of liver dysfunction, electroconvulsive therapy within the previous 6 months, diagnosis of possible or probable dementia or evidence of cognitive decline, history of Type I or Type II diabetes, history of significant CVD or myocardial infarction, cerebrovascular disease, pulmonary disease, cancer, untreated hypothyroidism, unstable or untreated hypertension, known osteoporosis or prior history of non-traumatic fracture, history of a neurological disorder or evidence of neurologic or other physical illness that could produce cognitive deterioration.

Forty-two medically stable men and women ages 23–71 with non-remitted depression were enrolled. Among enrolled participants, metabolic dysfunction ranged across the insulin sensitivity spectrum, including IS, IR and/or IR with hyperglycemia, for example, prediabetes.

Thirty-seven participants completed the study, including 9 males and 33 females. This includes 5 males and 17 females in the active arm, and 4 males and 16 in the placebo arm. Mean education of the whole group was 16.07 years, 15.91 for the active arm and 16.25 for the placebo arm. Five participants withdrew from the study; two moved, one withdrew due to a side effect (edema) of treatment with Pioglitazone and two did not specify a reason for withdrawal.

### Randomized controlled trial design and randomization

This randomized controlled trail consisted of a parallel design in which 50% of participants were allocated to 12 weeks of treatment with 30 mg per day of Pioglitazone and 50% of participants were randomized to 12 weeks treatment with placebo pill. Random allocation was generated by use of a random number generator that assigned half of the subjects' identification numbers to each study condition. A staff member of the Stanford University Department of Psychiatry and who did not participate in the implementation of the study performed randomization and maintenance of the unblinded study list. Active and placebo medication were bottled and labeled with participant identification numbers by this unblinded staff person according to randomized assignment. Study investigators, coordinators, raters and clinical laboratory staff remained blinded to subject treatment assignments throughout their participation in the study.

### Statistical analysis

Statistical analyses were conducted using Statistical Analysis System 9.4 (SAS Institute, Cary, NC, USA). Missing metabolic data, including telomere length, fasting plasma glucose and oral glucose tolerance test (OGTT) were imputed using fully conditional specification regression imputation.^27^ Hamilton score *T*-tests were conducted to assess baseline differences between treatment arms. Correlations and linear regression analysis were used to assess for the association between baseline telomere length and treatment response, including change in HDRS-21 and change in OGTT. Reliability of using change scores for the HDRS-21 scale was tested using the reliable change index.

### Clinical assessment

Clinical assessment consisted of a physical examination and laboratory tests, including measures of height and weight, an OGTT, fasting plasma glucose and fasting plasma insulin. Other data collected included current medications and family medical history. All clinical and laboratory tests were repeated at the end of treatment (Week 12) with the exception of the genetic sample.

### Oral glucose tolerance test

The OGTT measures individual ability to metabolize glucose and can distinguish between normal patterns and the patterns of diabetes and IR.^28^ We chose this test over other direct and surrogate measures of IR, such as the insulin suppression test or the hyperinsulinemic euglycemic clamp, because of its relatively short duration and participant tolerability. Furthermore, it does not involve administration of pharmacological agents, as do other assessments of glucose or insulin tolerance. Participants undergoing OGTT began in a fasting state (no food or drink except water for at least 10 h). Blood samples were obtained to measure baseline glucose and insulin concentrations, followed by administration of 75 mg of oral glucose, after which additional samples were obtained at +30, +60, +90 and +120 min. A meal was given to each participant immediately following completion of the procedure.

IR was defined by surrogate markers according to the following criteria:^29^

1. Fasting plasma glucose ⩾100 mg dl^−1^.
2. An OGTT at 120 min ⩾140 mg dl^−1^.
3. Fasting plasma insulin ⩾15 mIU/L.
4. Homeostatic model assessment-insulin resistance (HOMA-IR) >3.8.

### Psychiatric assessment

The psychiatric examination at screening included the Structured Clinical Interview for DSM-IV (SCID),^30^ the 17-item Hamilton Depression Rating Scale (HDRS-21)^31^ and the mini–mental state examination.^32^ Unremitted depression status was characterized by a 21-item HDRS (HDRS-21) score ⩾8 following at least 8 weeks of stable treatment-as-usual for their depression. We will not exclude subjects on the basis of a type of medication/s used in treatment of depression, as our primary goal is to evaluate antidepressant qualities of adjunctive Pioglitazone. Administration of the HDRS-21 was repeated at each interim visit (Weeks 2, 4, 6 and 8) and at the end of treatment (Week 12). The mini–mental state examination, a brief measure of global cognitive functioning was used to screen out current cognitive impairment. Among participants, 15 were identified as having 3 or fewer lifetime episodes of depression, 15 were identified as having 4 or more episodes and 12 subjects were unsure of the number of episodes. The mean duration of the current depressive episode was 397.21 days (s.d.=827.59). Twelve subjects were unable to estimate the duration of the current episode, or identified it as duration greater than 10 years. A board certified neuropsychologist who was blinded to the treatment assignment completed psychiatric tests and evaluations.

### LTL measurement

Total genomic DNA was purified using QIAamp DNA blood Mini kit (QIAGEN, Venlo, The Netherlands, Cat#51106) from whole blood stored at −80 °C and quantified by measuring OD260. The telomere length assay is adapted from the published original method by Cawthon.^33, 34^ The telomere thermal cycling profile consists of: cycling for T(telomic) PCR: 96 °C for 1 min; denature at 96 °C for 1 s, anneal/extend at 54 °C for 60 s, with fluorescence data collection, 30 cycles.

Cycling for S (single-copy gene) PCR: PCR: 96 °C for 1 min; denature at 95 °C for 15 s, anneal at 58 °C for 1 s, extend at 72 °C for 20 s, 8 cycles; followed by denature at 96 °C for 1 s, anneal at 58 °C for 1 s, extend at 72 °C for 20 s, hold at 83 °C for 5 s with data collection, 35 cycles.

The primers for the telomere PCR are *tel1b* (5′-CGGTTT(GTTTGG)_5_GTT-3′), used at a final concentration of 100 nM, and *tel2b* (5′-GGCTTG(CCTTAC)_5_CCT-3′), used at a final concentration of 900 nM. The primers for the single-copy gene (human beta-globin) PCR are *hbg1* (5′-GCTTCTGACACAACTGTGTTCACTAGC-3′), used at a final concentration of 300 nM, and *hbg2* (5′-CACCAACTTCATCCACGTTCACC-3′) used at a final concentration of 700 nM. The final reaction mix contains 20 mM Tris-HCl, pH 8.4; 50 mM KCl; 200 mM each dNTP; 1% DMSO; 0.4 × Syber Green I; 22 ng *Escherichia coli* DNA per reaction; 0.4 U of Platinum Taq DNA polymerase (Invitrogen, Waltham, MA, USA). To control for inter-assay variability, eight control DNA samples are included in each run. In each batch, the average telomere to single gene copy ratio T/S ratio of each control DNA is divided by T/S for the same DNA from 10 runs to get a normalizing factor.^34^ This is done for all eight samples and the average normalizing factor for all eight samples is used to correct the participant DNA samples to get the final T/S ratio. The T/S ratio for each sample was measured twice. When the duplicate T/S value and the initial value vary by >7%, the sample was run the third time and the two closest values were reported. The coefficient of variation for this study is 2.4%.) per 11 μl reaction; 6 ng of genomic DNA. Tubes containing 26, 8.75, 2.9, 0.97, 0.324 and 0.108 ng of a reference DNA (from the Hela cancer cell line) are included in each PCR run so that the quantity of targeted templates in each research sample can be determined relative to the reference DNA sample by the standard curve method. The same reference DNA was used for all PCR runs.^33^

## Results

Forty-two individuals with unremitted depressive disorder were evaluated for LTL at baseline. Their demographic and clinical characteristics are presented below (Table 1). LTL was not correlated with HDRS-21 at score baseline (*P*=0.29, *r*=0.17, 95% CI=−0.14,0.45). Neither did LTL differ between IR and IS subjects at baseline.

Baseline age and LTL were correlated at baseline assessment in the whole group (*P*=0.005, *r*=−0.42, 95% CI=−0.64,−0.13) and the active arm (*P*=0.003, *r*=−0.60, 95% CI=−0.81,−0.23), but not the placebo arm (*P*=0.93, *r*=−0.002, 95% CI=−0.46, 0.43). This may be due to the fact that subjects in the placebo group are significantly younger than subjects in the active arm (Table 1). In the whole sample, age and LTL were correlated in subjects older than 45 (*P*=0.04 *r*=−0.42, 95% CI=−0.70., −0.01), but not those younger than 45 (*P*=0.52, *r*=0.16, 95% CI=−0.33, 0.58).

**Figure 1:**
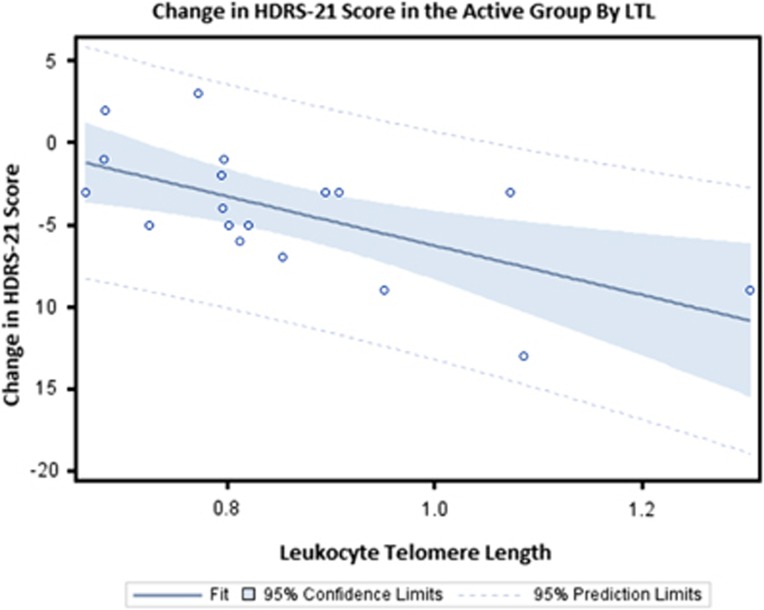
Change in HDRS-21 score in the active arm by LTL. HDRS-21, Hamilton Depression Rating Scale 21 Items; LTL, leukocyte telomere length.

Subjects with longer telomeres exhibited greater declines in depression severity in the active arm (*P*=0.005, *r*=−0.63, 95% CI=−0.84,−0.21) (Figure 1), but not in a placebo arm (*P*=0.36, *r*=0.27, 95% CI=−0.32, 0.69). There was no significant interaction between treatment arm and the LTL as predictors of HDRS-21 treatment response.

Conversely, LTL predicted improvement in OGTT in the group overall, and did not differ between intervention arms (*P*=0.036, *r*=−0.44, 95% CI=−0.74, 0.02) for the active arm, and *P*=0.026, *r*=−0.50, 95% CI=−0.78, −0.03) for placebo arm. There was no significant interaction between treatment arm and the LTL as predictors of OGTT treatment response.

Age and age group (dichotomized at 45 years) were added to generalized linear models to adjust for the potentially confounding effect of age on the relationship between LTL and HDRS-21or OGTT outcomes. Neither covariate was significant in any model. All associations in age and age-group-adjusted models remained significant, with the exception of the association between change in OGTT and LTL in the active group when adjusted for age (*β*=−59.01, *P*=0.14, 95% CI=−138.84, 20.82). However, this adjustment may be rendered inappropriate because age as a covariate was non-significant in the model (*β*=0.15, *P*=0.73, 95% CI=−0.73, 1.03).

## Discussion

The conceptual framework for the parent study was based on a model wherein treatment of underlying IR in patients with non-remitted depression will improve treatment outcome. In this report, we assessed LTL as a predictor of treatment outcome.

The main finding in this study was significant association between the LTL and antidepressant response in active arm, but not in placebo arm in persons with unremitted depression. While there were no differences in LTL between IR and IS subjects at baseline, LTL strongly predicted change in insulin sensitivity in both active and placebo arm. While the correlation between LTL and change in depression severity, as well as correlation with baseline IR was significant, the strength of correlation was stronger between LTL and antidepressant response. The lack of differences between active and placebo arms for changes in IR suggests that LTL predicts an improvement not specific to the insulin-sensitizing mechanism, but possibly another, for example, anti-inflammatory mechanism of PPAR-γ agonist. Contrary to our non-significant findings, a significant negative correlation was reported between LTL and HOMA-IR in patients with impaired glucose tolerance^35^ and newly diagnosed type 2 diabetes.^36^ It is possible that this difference is accounted for by the fact that our subjects were at earlier stages of IR, and that negative correlation becomes apparent when glucose dysregulation becomes more advanced.

Two independent risk factors for accelerated aging telomere shortening and IR have been associated with chronic depressive illness.^14, 37^ The role of LTL in relation to depressive disorders has been reviewed in ref. 12 with some^36, 38^ but not all^39, 40^ studies suggesting reduced LTL in treated and untreated depressed patients. Unlike some larger studies,^9^ but similar to some other studies,^26, 41^ we did not find an association between depression severity and LTL. Unlike Wolkowitz *et al.*^37^ and Verhoeven *et al.*,^9^ we did not find a negative association between LTL and chronicity of depressive disorder. However, our sample was smaller, and while Verhoeven *et al.*^9^ assessed chronicity via duration of depressive episode, this study assessed number of episodes longer than 6 months in duration.

Similar to our results, Wolkowitz *et al.*^42^ found an association between telomerase and antidepressant response to psychotropic agents. We observed an inverse association between baseline LTL and change in HDRS-21 score in the active (PPAR-γ agonist), but not in placebo arm. To the contrary, Hartmann *et al.*^26^ found no correlation between LTL and response to treatment of depression. The difference between the Hartmann study and ours may be due to the choice of treatment agents (antidepressants vs PPAR-γ agonist), duration of treatment and study design (open label vs placebo controlled).

Understanding the role of biomarkers in accelerated aging for prediction of response to treatment of depression is based on a fundamental biological premise of shortened life span as a result of environmental insults such as stress, major somatic and psychiatric illnesses. Among the most common mediators of accelerated aging in depressive disorders is metabolic dysfunction, for example, IR, inflammation and oxidative stress. Chronic exposure to inflammatory cytokines, oxidative stress and glucocorticoids may also accelerate telomere shortening.^37^ IR is a proinflammatory state and is in turn associated with oxidative stress. Our results suggest that both IR and inflammation may mediate an antidepressant response to PPAR-γ agonist, as both IR and IS at baseline subjects exhibited improvement in depression severity.^19^

A major limitation was the small sample size, rendering these findings preliminary. The innovative choice of therapeutic agent, placebo-controlled design and well-described IR are among unique features of this study. Our results augment current understanding of the mechanisms of antidepressant response, and, if replicated in larger samples, will aid in predicting treatment outcome in a depressive disorder.

## Figures and Tables

**Table 1:** Baseline demographic characteristics by treatment arm

	*Active (*N=*22)*		*Placebo (*N=*20)*		t*-test of group differences (*N=*42)*	
	*Mean*	*95% CI*	*Mean*	*95% CI*	t	P
Age	49.42	42.14–56.70	43.28	37.41–49.14	1.61	0.12
BMI	29.98	27.82–33.84	30.04	27.44–35.16	−0.19	0.85
Fasting plasma glucose^a^	97.25	90.71–103.40	96.90	93.82–102.57	0.10	0.92
Leukocyte telomere length^a^	0.87	0.79–0.95	0.99	0.93–1.08	−2.48	**0.02**
HOMA^a^	3.14	2.00–3.58	3.11	2.45–4.38	0.05	0.96
Oral glucose tolerance test^a^	103.88	91.23–110.62	131.6	107.07–166.43	−2.07	**0.05**
Baseline HDRS-21	16.73	14.60–19.93	14.55	8.25–14.09	1.43	0.16

Abbreviations: BMI, body mass index; CI, confidence interval.

a Missing data were imputed using fully conditional specification regression. ^b^Bolded values significant at *P*<0.05.
